# Enhancement of Protective Efficacy through Adenoviral Vectored Vaccine Priming and Protein Boosting Strategy Encoding Triosephosphate Isomerase (SjTPI) against *Schistosoma japonicum* in Mice

**DOI:** 10.1371/journal.pone.0120792

**Published:** 2015-03-20

**Authors:** Yang Dai, Xiaoting Wang, Jianxia Tang, Song Zhao, Yuntian Xing, Jianrong Dai, Xiaolin Jin, Yinchang Zhu

**Affiliations:** 1 Key Laboratory of Parasitic Disease Control and Prevention, Ministry of Health and Jiangsu Provincial Key Laboratory of Parasite Molecular Biology, Jiangsu Institute of Parasitic Diseases, Wuxi, Jiangsu, China; 2 Jiangsu Stem Cell Key Laboratory, Institute of Medical Biotechnology, Medical College of Soochow University, Suzhou, Jiangsu, China; Federal University of São Paulo, BRAZIL

## Abstract

**Background:**

Schistosomiasis japonica is a zoonotic parasitic disease; developing transmission blocking veterinary vaccines are urgently needed for the prevention and control of schistosomiasis in China. Heterologous prime-boost strategy, a novel vaccination approach, is more effective in enhancing vaccine efficacy against multiple pathogens. In the present study, we established a novel heterologous prime-boost vaccination strategy, the rAdV-SjTPI.opt intramuscular priming and rSjTPI subcutaneous boosting strategy, and evaluated its protective efficacy against Schistosoma japonicum in mice.

**Methodology/Principal Findings:**

Adenoviral vectored vaccine (rAdV-SjTPI.opt) and recombinant protein vaccine (rSjTPI) were prepared and used in different combinations as vaccines in a mouse model. The specific immune responses and protective efficacies were evaluated. Furthermore, the longevity of protective efficacy was also determined. Results showed that the rAdV-SjTPI.opt priming-rSjTPI boosting strategy elicited higher levels of specific IgG responses and broad-spectrum specific cellular immune responses. The protective efficacy could reach up to nearly 70% and 50% of protection could be observed at 10 weeks after the last immunization in mice.

**Conclusions/Significance:**

The rAdV-SjTPI.opt intramuscular priming-rSjTPI subcutaneous boosting vaccination strategy is a novel, highly efficient, and stable approach to developing vaccines against Schistosoma japonicum infections in China.

## Introduction

Schistosomiasis, one of the neglected tropical diseases caused by intravascular trematodes of the genus *Schistosoma*, is still one of the world’s major public health problems [1, 2]. It is endemic in 78 countries or regions from Africa, Asia to Southern America. Until 2012, at least 249 million people required preventive treatment for schistosomiasis [3]. In China, schistosomiasis caused by *Schistosoma japonicum*, is one of the most prevalent parasitic diseases. Despite efficient control for many years, schistosomiasis remains endemic in the lowland marsh areas or lake regions of Hunan, Hubei, Jiangxi, Anhui, and Jiangsu provinces and in the mountain areas of Sichuan and Yunnan provinces [4, 5]. By the end of 2012, it was estimated that there were 240,597 cases of schistosomiasis japonica distributed throughout 452 counties and 1,189,829 cattle raised in epidemic areas [6].

Praziquantel-based chemotherapy plays an important role in the process of schistosomiasis control for its therapeutic effectiveness, safety, and low cost [7]. However, praziquantel treatment does not prevent reinfection and may require repeated chemotherapy [8]. Repeated mass chemotherapy treatment may generate drug resistance or decreased effectiveness against worms [9, 10]. Moreover, schistosomiasis japonica is a zoonotic disease, and animal reservoir hosts, including water buffalo, cattle, pigs, and goats, play an important contribution to sustaining the infection [11, 12]. Therefore, developing transmission blocking veterinary vaccines is urgently needed for the prevention and control of schistosomiasis in China.

Vaccines against *Schistosoma japonicum* infection have been studied for decades. Numerous antigen candidates from all stages of the *Schistosoma japonicum* life cycle were indentified, including 23 kDa membrane protein (Sj23), fatty acid binding protein (SjFABP), triosephosphate isomerase (SjTPI), and glutathione-S-transferase (SjGST). However, the worm reduction rates induced by these antigens in mice were below or not stabilized at 50% levels, values recommended by the World Health Organization (WHO) [13–17]. Therefore, to improve the protective efficacy is the predominant issue for vaccine development against *Schistosoma japonicum* infection.

Heterologous prime-boost vaccination strategy, which immunizes through unmatched vaccine delivery methods while using the same antigen, is more effective than the traditional vaccination strategy, homologous prime-boost, and is considered a novel vaccination approach [18]. The strategy has been widely used in vaccine research against malaria, tuberculosis, and AIDS, with different prime-boost formats such as DNA priming and protein boosting, DNA priming and viral based vaccine boosting, etc. [19–21]. In schistosome vaccine research, we made an attempt to improve the efficacy with a DNA priming and protein boosting strategy previously, and results showed that the worm reduction rate could be elevated from 26.9% or 32.88% (DNA) to 36.9% or 45.35%, respectively[22, 23].

SjTPI, a potential vaccine candidate, has been studied for many years [24, 25]. In our previous study, we constructed a replication-defective, adenoviral-based vaccine encoding the optimized SjTPI gene (rAdV-SjTPI.opt) which, when injected intramuscularly into mice, could elicit a high level of specific Th1, IgG responses and a 50% worm reduction rate [26]. To further improve the protective efficacy induced by rAdV-SjTPI.opt, in the present study, we immunized mice with the rAdV-SjTPI.opt intramuscular priming and rSjTPI protein subcutaneous boosting strategy, tested the specific immune responses, and evaluated the protective efficacy through challenge infection of *Schistosoma japonicum* with cercariae. Furthermore, the longevity of specific immune responses and protective efficacy induced by this strategy was also evaluated in the mice model.

## Materials and Methods

### Ethics statement

Animal experiments were performed in accordance with the guideline for administration of lab animals, issued by the Ministry of Science and Technology (Beijing, China). Mice were housed in a 12 hour/12 hour light/dark cycled barrier system and fed with sterilized food and water. All efforts were made to alleviate suffering, including anesthesia mice with 1% pentobarbital sodium solution (60 mg/kg) when undergoing immunization, monitoring mice in every week after immunization or infection. All procedures relevant to the treatment of animals were approved by the Institutional Review Board (IRB00004221) of Jiangsu Institute of Parasitic Diseases (Wuxi, China).

### Parasites

*Oncomelania hupensis* infected with *S*. *japonicum* were provided by the Jiangsu Institute of Parasitic Diseases (Wuxi, China). The cercariae were collected from infected snails and used for challenge.

### Preparation of vaccines

Recombinant proteins (rSjTPI) were purified from a prokaryotic expression system (pGEX-4T-3 as a vector, constructed previously), using the Bulk and Redipack GST purification modules (GE Healthcare) [27], and thrombin (Sigma, Santa Clara, USA) was used for removing the GST-tag. The rSjTPI was diluted with PBS to a final concentration of 1.0mg/ml, stored in aliquots at −80°C and emulsified with equal volume of Freund’s adjuvant (Sigma, Santa Clara, USA) before immunization. The mixture was used as protein vaccine. Recombinant adenoviral vectored vaccines (rAdV-SjTPI.opt) were constructed and purified in our previous study [26]. Adenoviral vectored vaccines and vectors were stored in aliquots at −130°C till use.

### Animal immunization

Female six-week-old BALB/c mice were purchased from SLAC laboratory animal center (Shanghai, China) and used for vaccination studies. The mice were randomly divided into five different groups (16 mice / group), including the rAdV-SjTPI.opt (immunized intramuscularly, i.m.) priming-rSjTPI (immunized subcutaneously, s.c.) boosting group (rAdV-SjTPI.opt + rSjTPI), rAdV-SjTPI.opt i.m. group (rAdV-SjTPI.opt), rSjTPI s.c. group (rSjTPI), Ad vector i.m. group (Ad vector), and blank control group (Control, without any immunization). The heterologous prime-boost group was immunized 4 times (3 times for priming and one for boosting); the other groups were immunized 3 times. The i.m. and s.c. immunization procedures were performed according to our previous studies [22, 26]. Each mouse was immunized every 2 weeks with 1×10^8^ pfu adenovirus or 100 μg protein vaccine. In rSjTPI group, Complete Freund’s adjuvant was used in the first immunization and Incomplete Freund’s adjuvant was used in the second and third immunization. However, in rAdV-SjTPI.opt priming-rSjTPI boosting group, Incomplete Freund’s adjuvant was used in protein-boosting immunization.

### rSjTPI-specific IgG assay

Serum samples of each mouse were collected from their caudal veins before immunization and parasite challenge. Enzyme linked immunosorbent assays (ELISAs) were performed to detect specific IgG responses, including IgG, IgG subclasses (IgG1 and IgG2a) levels, IgG avidity, and IgG titer. The recombinant protein, rSjTPI, was used as the antigen source. For IgG and IgG subclass detection, ELISA plates (Nunc) were coated with rSjTPI (0.2 μg/well) and serum samples were tested with a 1:100 dilution. HRP-conjugated goat-anti-mouse IgG, IgG1, and IgG2a (SouthernBio; Birmingham, USA) were diluted at 1:5000. The optical density (OD) was read at 450 nm with a Microplate Reader (Antobio; Zhengzhou, China). For IgG avidity detection, following the serum incubation step, an additional washing step with 6 M urea in PBST was performed to discard the low avidity IgG, and the avidity index was calculated as the ratio of the OD_450_ treated and OD_450_ untreated, according to the published references[28,29]. For IgG titer detection serum samples were examined using multiple dilutions (from 1:50 to 1,638,400) for each mouse. The IgG titer of each sample was determined by comparison with the OD_450_ value of the control (cut-off value ≥2.1× the mean OD_450_ value of control).

### Cytokine levels determination

Two days before challenge, 4 mice from each group were sacrificed randomly and the single-cell suspension was prepared. The splenocytes (5×10^5^ cells per well) were cultured in triplicate for each mouse in 96-well plates (Corning, NewYork, USA). Cells were incubated in RPMI 1640 medium (Hyclone, USA), supplemented with 10% fetal calf serum (Gibco, USA), and stimulated with rSjTPI (10 μg/ml), ConA (Sigma, 10 μg/ml), or media alone at 37°C with 5% CO_2_ for 72 h. Cytokine levels in the supernatant were measured using flow cytometry analysis (BD FACSCalibur Flow Cytometer; BD, USA) and the BD Cytometric Bead Array (CBA) Mouse Th1/Th2/Th17 Cytokine Kit, according to the manufacturer’s protocols.

### Elispot assay

The amount of IL-4 and IFN-γ secreting cells were determined using the Mouse IL-4 and IFN-γ ELISpot kits (R&D, USA), according to the manufacturer’s instructions. The procedures of single-cell suspension preparation and stimulation were in accordance with the above-mentioned instructions. Spot forming units (SFU) were counted using the ELISpot ImmunoSpot S5 Analyzer (C.T.L., Germany), and analyzed using the C.T.L. ImmunoSpot image software (Version 5.1). The results were expressed as SFU per 1×10^6^ cells.

### Challenge and efficacy evaluation

Two weeks after the last vaccination, each mouse was challenged with 40±1 *S*. *japonicum* cercariae by abdominal skin penetration. Six weeks post challenge, all mice were sacrificed and the worm and egg burden was determined. The total worm burden (male and female) were determined by counting the number of adult worms recovered from the portal vein. The reduction rate was calculated with the following formula: reduction rate = (1 − average total worm or female worm burden in the test group / average total worm or female worm burden in the control group) ×100%.The liver egg numbers were determined by weighing the whole liver and then digesting the liver with 5 ml 5% KOH at 37°C for 48–72 h. The liver egg reduction rate was calculated using the following formula: liver egg reduction rate = (1 − number of eggs per gram liver in the test group / number of eggs per gram liver in the control group) ×100%.

### Histopathologic examination in liver

The area of a single egg granuloma in the liver was observed using sectioned liver tissue (1–5 cm^3^) from each mouse. The sections were prepared according to standard histological procedures, including fixed in 4% formaldehyde, dehydrated in the alcohol, embedded in paraffin and stained with hematoxylin-eosin. Egg granulomas in the liver were examined and photographed under a light microscope (Olympus BX51, Japan) and at least 15 single egg granulomas per group from each mouse were photographed. The area of each single-egg granuloma was determined using a computerized image analysis system (JD801 Version 1.0, China). Granuloma sizes were expressed as means of areas measured in μm^2^ ± SD.

### Evaluation of protective longevity induced by rAdV-SjTPI.opt priming-rSjTPI boosting strategy

The protective longevity of rAdV-SjTPI.opt priming-rSjTPI boosting vaccination strategy was evaluated in the following animal study. Six-week-old female BALB/c mice were randomly divided into two groups (45 mice / group), including control group and rAdV-SjTPI.opt priming-rSjTPI boosting group. The immunization procedure was consistent with the above mentioned. The mice (10 mice each time) were challenged with 40±1 *S*. *japonicum* cercariae at 2, 6, and 10 weeks after the last immunization. Serum samples from each group were collected before immunization and challenge, and used for specific IgG detection. The supernatant of stimulated single-cell suspension in each group was prepared (5 mice/group each time) and used for cytokine analysis. The specific protective efficacies were also detected. All procedures were in according with the above-mentioned instructions.

### Statistical analysis

Statistical analysis was performed using the SPSS software (Version 19.0). A one-way analysis of variance (ANOVA) was used for data comparison between different groups, and Bonferroni method was used for comparison between any two means. *P* values <0.05 were considered statistically significant.

## Results

### 1. Levels of rSjTPI-specific IgG responses induced by different immunizations

**Fig 1:**
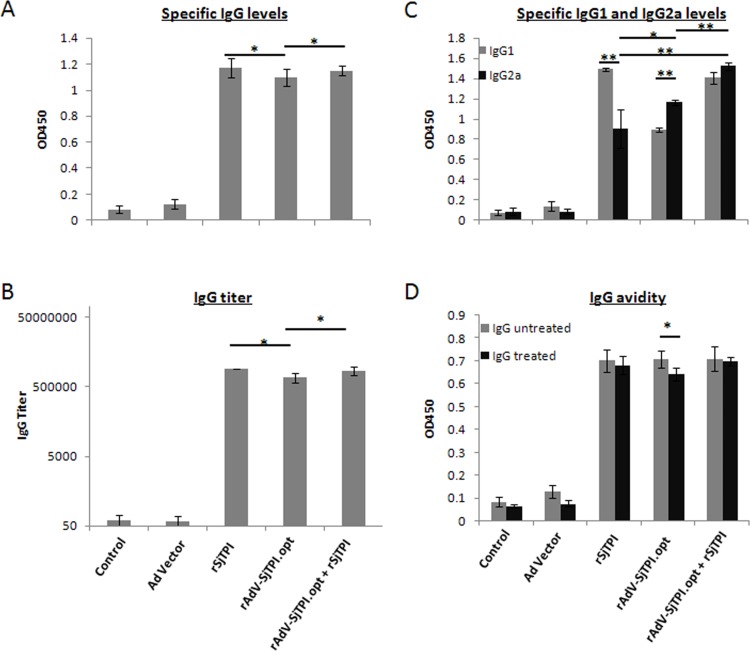
rSjTPI-specific antibody responses in mice sera induced by Ad vector, rSjTPI, rAdV-SjTPI.opt, rAdV-SjTPI.opt priming-rSjTPI boosting immunized groups, and control group. (A) IgG responses; (B) IgG titer; (C) IgG1 and IgG2a responses; (D) IgG avidity; each bar represents the mean ± standard deviation (SD). *P<0.05; **P<0.01.

Compared with the control group, the rAdV-SjTPI.opt, rSjTPI, and rAdV-SjTPI.opt priming-rSjTPI boosting immunizations elicited a higher IgG level, IgG titer and avidity, but the rSjTPI and the rAdV-SjTPI.opt priming-rSjTPI boosting immunizations elicited higher IgG responses (including IgG levels, IgG titer, and avidity) than rAdV-SjTPI.opt immunization (see Fig. 1A, 1B and 1D).The IgG avidity indexes of the three groups were 0.973, 0.809 and 0.983, respectively. Different IgG subclass levels were induced by the three immunization types (see Fig. 1C). The rSjTPI immunization induced a higher IgG1 level, with a IgG2a/IgG1 ratio of 0.61. The rAdV-SjTPI.opt immunization induced a higher IgG2a level, with a IgG2a/IgG1 ratio of 1.31. The IgG1 and IgG2a levels were elicited simultaneously in the rAdV-SjTPI.opt priming-rSjTPI boosting group, with an IgG2a/IgG1 ratio of 1.08. Furthermore, therAdV-SjTPI.opt priming-rSjTPI boosting immunization elicited the highest specific IgG2a levels.

### 2. rAdV-SjTPI.opt priming-rSjTPI boosting strategy induced broad spectrum specific cellular immune responses

**Fig 2:**
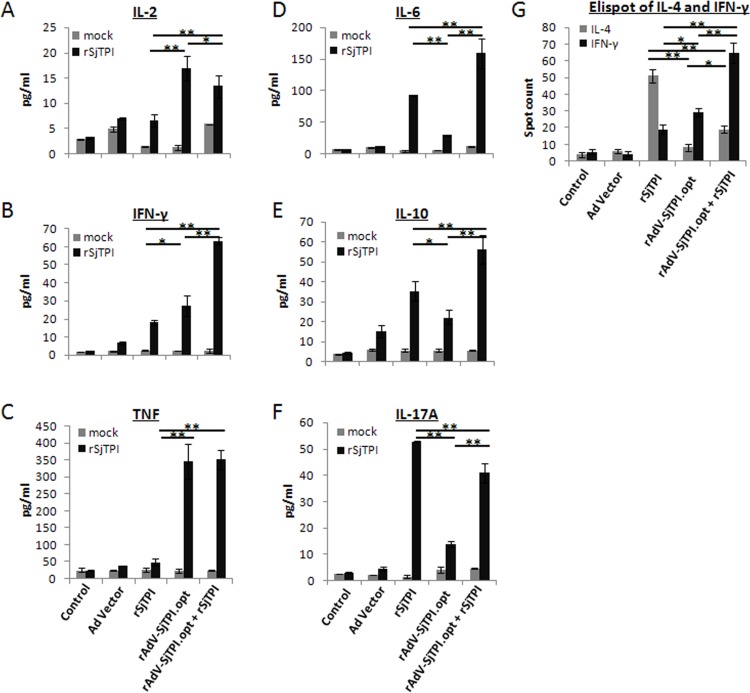
rSjTPI-specific cytokine responses in splenocytes induced by Ad vector, rSjTPI, rAdV-SjTPI.opt, rAdV-SjTPI.opt priming-rSjTPI boosting immunized groups, and control group. Panels A-F show IL-2, IFN-γ, TNF, IL-6, IL-10, and IL-17A levels, respectively, in splenocyte culture supernatants stimulated with rSjTPI (10 μg/ml) or medium (mock); the splenocytes were also stimulated with rSjTPI (10 μg/ml). Panel G shows the spot counts of IL-4 and IFN-γ secreting cells in each group. Each bar represents the mean ± standard deviation (SD). *P<0.05; **P<0.01.

To evaluate specific cellular immune responses induced by vaccines, Th1, Th2, and Th17 type cytokines produced by splenocytes were detected through CBA and Elispot methods. Results showed that splenocytes from mice immunized with rAdV-SjTPI.opt produced higher levels of Th1 cytokines (IL-2, TNF, and IFN-γ) than that immunized with Ad vector (see Fig. 2A-C). Similar results were observed with the number of IFN-γ secreting cells (see Fig. 2G). Subcutaneous rSjTPI immunization induced higher levels of Th2 (IL-4, IL-6, and IL-10) and Th17 (IL-17A) cytokines than the control group (Fig. 2D-G). However, splenocytes from mice immunized with the rAdV-SjTPI.opt priming-rSjTPI boosting strategy produced higher levels of Th1, Th2, and Th17 cytokines than control group. Furthermore, the IFN-γ, IL-6 and IL-10 levels elicited with the rAdV-SjTPI.opt priming-rSjTPI boosting strategy were higher than those generated in the rAdV-SjTPI.opt or rSjTPI immunization group; and the IL-17A levels in the rAdV-SjTPI.opt priming-rSjTPI boosting group were higher than in the rAdV-SjTPI.opt group but lower than in the rSjTPI group. No IL-4 was detected by CBA (data not shown). These findings suggest that the rAdV-SjTPI.opt priming-rSjTPI boosting strategy induced a broader spectrum specific cellular immune response.

### 3. rAdV-SjTPI.opt priming-rSjTPI boosting strategy synergistically enhanced the protective efficacy against *Schistosoma japonicum* challenge

**Fig 3:**
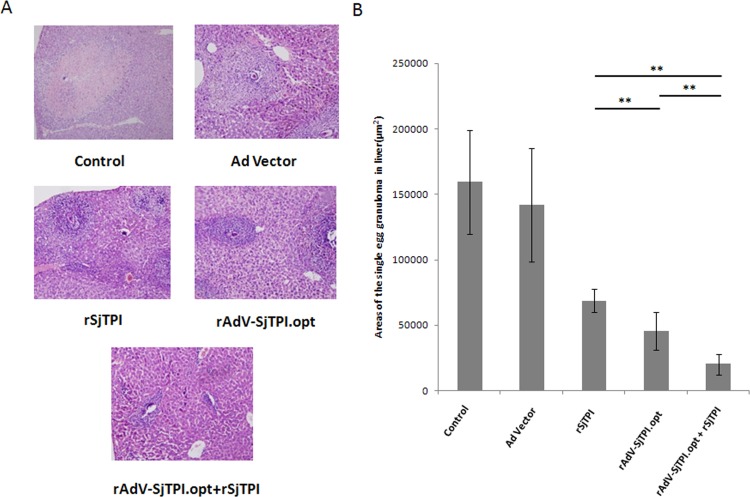
The single egg granuloma responses in liver induced by Ad vector, rSjTPI, rAdV-SjTPI.opt, and rAdV-SjTPI.opt priming-rSjTPI boosting immunization. (A) Representative granuloma of each group, induced by a single egg in liver (magnification factor 10×10); (B) Areas of the single egg granuloma in liver. Data is presented as the mean ± standard deviation (SD). *P<0.05; **P<0.01.

Results of protective efficacy in each group were shown in Fig. 3 and Table 1. Compared with the control group and the Ad vector immunization, the rAdV-SjTPI.opt, rSjTPI, and rAdV-SjTPI.opt priming-rSjTPI boosting groups resulted in lower numbers of adult and female worms and eggs in the liver (see Table 1). Worm and egg burdens in the liver were significantly lower after immunization with rAdV-SjTPI.opt than after immunization with rSjTPI (see Table 1). The number of worms and eggs in the liver in the rAdV-SjTPI.opt priming-rSjTPI boosting group were significantly lower than that in the rSjTPI and rAdV-SjTPI.opt groups. The area of single egg granuloma in the rAdV-SjTPI.opt, rSjTPI and rAdV-SjTPI.opt priming-rSjTPI boosting groups was smaller than that in the Ad vector or control group. The area of single egg granuloma was smaller in the rAdV-SjTPI.opt group than in the rSjTPI group. However, the smallest area of single egg granuloma was observed in the rAdV-SjTPI.opt priming-rSjTPI boosting immunization group (see Fig. 3A and B). These findings suggest that the rAdV-SjTPI.opt priming-rSjTPI boosting vaccination strategy could synergistically enhance the protective efficacy against *Schistosoma japonicum* in a challenge infection of mice.

**Table 1:** Summary of the protective efficacies of the different immunization groups.

Group	No. of mice	Adult worms		Female worms		Eggs in liver	
		No. of worms	Reduction (%)	No. of worms	Reduction (%)	No. of eggs	Reduction (%)
Control	11	28±3	—	14±2	—	114434±17170	—
Ad vector	12	27±3	6.47	13±2	4.88	106826±18808	6.65
rSjTPI	12	21±5^#^	26.67	10±2^#^	25.20	70993±28772^#^	37.96
rAdV-SjTPI.opt	12	14±5^#^ ^,^ *	50.59	6±2^#^ ^,^ *	54.77	54883±26892^#^ ^,^ *	52.04
rAdV-SjTPI.opt + rSjTPI	12	8±2^#^ ^,^ * ^,^ ^&^	72.09	4±1^#^ ^,^ * ^,^ ^&^	72.73	31891±17776^#^ ^,^ * ^,^ ^&^	72.13

^#^ Statistically significant (P<0.01), compared with the control or vector control group.

* Statistically significant (P<0.01), compared with the rSjTPI group.

^&^ Statistically significant (P<0.01), compared with the rSjTPI or rAdV-SjTPI.opt group.

### 4. Longevity of specific immune responses induced by rAdV-SjTPI.opt priming-rSjTPI boosting vaccination strategy

**Fig 4:**
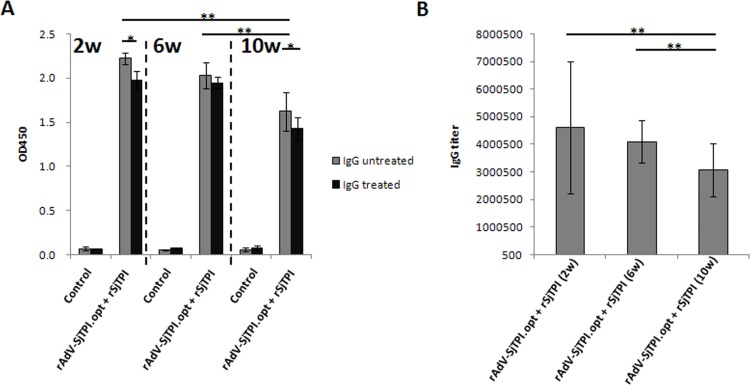
rSjTPI-specific IgG responses induced by rAdV-SjTPI.opt priming-rSjTPI (s.c.) boosting strategy at 2, 6, and 10 weeks after the last immunization. Panel A shows IgG responses and IgG avidity. Panel B shows the IgG titers. Data is presented as the mean ± standard deviation (SD). *P<0.05; **P<0.01.

Compared with the control, rAdV-SjTPI.opt priming-rSjTPI boosting strategy immunization elicited higher specific IgG responses (including IgG level, IgG avidity, and IgG titer) at 2, 6, and 10 weeks after the last immunization (see Fig. 4A and B). However, a significant decrease of specific IgG levels and IgG titers were observed after 10 weeks after the immunization, compared with 2 or 6 weeks after the last immunization. There was no significant difference of specific IgG levels and IgG titers between 2 and 6 weeks after the last immunization. A higher specific IgG avidity was observed at 6 weeks after the last immunization and the avidity indexes of the 3 time points observed were 0.888, 0.958, and 0.882, respectively.

**Fig 5:**
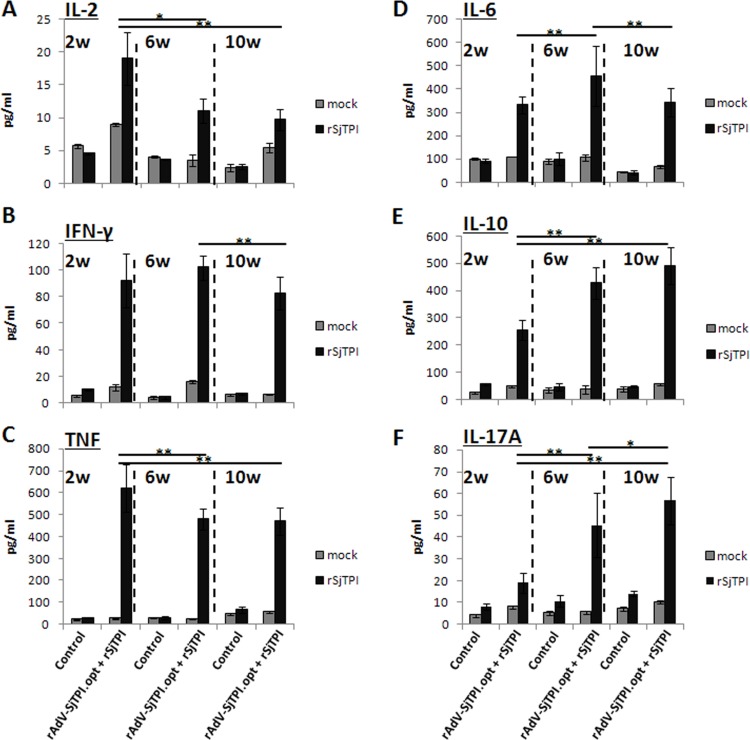
rSjTPI-specific cytokine responses induced by rAdV-SjTPI.opt priming-rSjTPI boosting strategy at 2, 6, and 10 weeks after the last immunization. Panels A-F show IL-2, IFN-γ, TNF, IL-6, IL-10, and IL-17A levels, respectively, in splenocyte culture supernatants stimulated with rSjTPI (10 μg/ml) or medium (mock). Data is presented as the mean ± standard deviation (SD). *P<0.05; **P<0.01.

Specific cytokines produced by splenocytes were detected with the CBA method. Results showed that this vaccination strategy elicited higher levels of Th1 (IL-2, IFN-γ and TNF), Th2 (IL-6 and IL-10), and Th17 (IL-17A) cytokines than the control at 2, 6 and 10 weeks after the last immunization (see Fig. 5). Splenocytes from the rAdV-SjTPI.opt priming-rSjTPI boosting immunization produced higher IL-2 and TNF levels at 2 weeks than that at 6 and 10 weeks after the last immunization, and there was no significant difference between 6 and 10 weeks after immunization (Fig. 5A and C). A significant higher IFN-γ level was observed at 2 and 6 weeks after immunization (see Fig. 5B). A higher IL-6 level was observed at 6 weeks after immunization and there was no significant difference between 2 and 10 weeks after immunization (see Fig. 5D). In the rAdV-SjTPI.opt priming-rSjTPI boosting group, the IL-10 level at 2 weeks after immunization was lower than that at 6 or 10 weeks after immunization, and there was no significant difference between 6 and 10 weeks after immunization (see Fig. 5E). The IL-17A level was increased gradually and the highest level was observed at 10 weeks after immunization (see Fig. 5F).No IL-4 was detected with CBA (data not shown).

### 5. Longevity of the protective efficacy induced by rAdV-SjTPI.opt priming-rSjTPI boosting vaccination strategy against *Schistosoma japonicum* infection

Compared with the control group, the rAdV-SjTPI.opt priming-rSjTPI boosting strategy led to a lower number of adult worms, female worms, and eggs in the liver at 2, 6, and 10 weeks after the last immunization, respectively (see Table 2). In the rAdV-SjTPI.opt priming-rSjTPI boosting group, the adult and female worm burdens at 2 or 6 weeks after the last immunization were significantly lower than that at 10 weeks after the last immunization, and there was no significant difference between 2 and 6 weeks after immunization. However, the egg burden at 2 weeks after immunization was significantly lower than that at 6 or 10 weeks after immunization in the rAdV-SjTPI.opt priming-rSjTPI boosting group, and there was no significant difference between 6 and 10 weeks after immunization. These findings suggest that the rAdV-SjTPI.opt priming-rSjTPI boosting strategy could have elicited a high and stable protective efficacy against *Schistosoma japonicum* in challenge infections of mice, and 50% of protection could be observed at even 10 weeks after the last immunization.

**Table 2:** Summary of the protective efficacies induced by rAdV-SjTPI.opt priming-rSjTPI s.c. boosting vaccination strategy at different challenge time points.

Group	Challenge time	No. of mice	Adult worms		Female worms		Eggs in liver	
			No. of worms	Reduction (%)	No. of worms	Reduction (%)	No. of eggs	Reduction (%)
Control	2w	10	25±2	—	13±1	—	52557±16623	—
	6w	10	30±2	—	15±1	—	59997±10037	—
	10w	9	28±3	—	14±2	—	59762±12904	—
rAdV-SjTPI.opt + rSjTPI	2w	10	9±2^#^ ^,^ *	66.01	4±1^#^ ^,^ *	69.52	17042±4240^#^	67.57
	6w	10	11±2^#^ ^,^ *	63.48	5±1^#^ ^,^ *	63.93	25786±3427^#^ ^,^ ^&^	57.02
	10w	10	15±3^#^	48.83	7±1^#^	50.39	27950±4126^#^ ^,^ ^&^	53.23

^#^ Statistically significant (P<0.01), compared with the control group at different challenge time point.

*Statistically significant (P<0.05), compared with the rAdV-SjTPI.opt priming-rSjTPI boosting strategy at 10 weeks after the last immunization.

^&^ Statistically significant (P<0.05), compared with the rAdV-SjTPI.opt priming-rSjTPI boosting strategy at 2 weeks after the last immunization.

## Discussion

We established a novel heterologous prime-boost vaccination strategy, the rAdV-SjTPI.opt intramuscular priming and rSjTPI subcutaneous boosting strategy, and evaluated the protective efficacy against *Schistosoma japonicum* in mice. Results showed that this strategy induced a higher level of specific IgG responses and broad spectrum specific cellular immune responses. The protective efficacy could reach up to nearly 70% and 50% of protection could be observedat 10 weeks after the last immunization in mice.

Heterologous prime-boost strategies based on different combinations have emerged as a powerful approach in various infectious models to improving specific immune responses and to increasing the efficacy in challenge infections [30, 31]. The underlying mechanisms of heterologous prime-boost vaccination are still unclear till now. Evidences showed that the higher efficacy of heterologous prime-boost vaccination might be associated with the elicited high avidity antibodies, broad spectrum of specific immune responses, and the circumvention of anti-vector effect [32, 33]. In the present research, a synergistic effect was obtained when mice were immunized with the rAdV-SjTPI.opt intramuscular priming and rSjTPI subcutaneous boosting strategy; manifested as enhancement of protective efficacy, augmentation of the specific IgG and T cell responses, and expansion of the immune response spectrum.

Replication deficient adenoviral vectors retain the ability to actively invade target cells and show high transfection efficiency when used as a vaccine delivery system [34, 35]. Adenoviral based vaccines injected intramuscularly could express the antigen efficiently in muscular cells which present the antigen through the MHC-I processing pathway. However, recombinant protein vaccines injected subcutaneously could be swiftly engulfed by antigen presenting cells and be present through the MHC-II antigen processing pathway. Different antigen processing pathways may selectively activate different T cell subclasses and enhance cooperation between T and B cells [36–38]. In the current study, we observe that the rAdV-SjTPI.opt intramuscular priming and rSjTPI subcutaneous boosting strategy, induced a stronger and a broader spectrum immune response than rAdV-SjTPI.opt or rSjTPI immunized solely (homologous prime-boost immunization strategy). This phenomenon may be due to the different antigen processing pathways of the two different vaccine delivery methods.

Previous studies suggest that high level of specific IFN-γ and IgG2a responses were associated with a high degree of protection against *Schistosoma japonicum* infection in mice and pigs [39–41]. However, specific Th2 responses may be more effective to elicit high protection in water buffaloes [42]. In this study, the protective efficacy elicited by rAdV-SjTPI.opt priming-rSjTPI boosting strategy was reproducible in mice through two independent animal experiments. High levels of IFN-γ and IgG2a responses were induced by this strategy and ultimately led to a high worm reduction rate in mice (>50%). Furthermore, simultaneously, a highly specific Th2 response was induced by this strategy. These results led to important information which will allow us to evaluate protective efficacy in large animal models in the near future. We also observed that reduced IL-17A levels were associated with relative high protective efficacy in the present research. This finding was consistent with our previous study; reduced IL-17A may partially contribute to improve the protective efficacy against *Schistosoma japonicum* infection [26, 43].

Protective lasting time is an important indicator for vaccine development. Schistosomiasis japonica, a seasonal epidemic disease, is mainly endemic in summer and fall (from May to October), in China [44]. To evaluate the protective longevity of the rAdV-SjTPI.opt priming-rSjTPI boosting strategy, we challenged mice at 2, 6, and 10 weeks after the last immunization. Data showed that the protective efficacy was stable at approximately 70% level at 2 and 6 weeks, but decreased to approximately 50% level at 10 weeks in mice. This result was contrary to the previous report using heterologous prime-boost strategy which could elicit long lasting protection against other infectious diseases [45, 46]. A reason for the difference might be due to the different animal models used. In the current study, female BALB/c mice were used to evaluate the protective lasting time. It was reported that the splenocyte proliferation and IL-2 inducing ability of BALB/c mice were decreased significantly when the mice were aged over 5 months [47, 48]. This age factor may explain the significant decrease of protection at 10 weeks after the last immunization (5.5 months for actual age). Furthermore, different pathogen may be another important factor for this. However, data obtained from the present study was still valuable, and the protective efficacies induced by the V+V+V+P strategy will be evaluated in large animals (such as goat and water buffalo) in the near future.

In conclusion, we found that the rAdV-SjTPI.opt intramuscular priming and rSjTPI subcutaneous boosting vaccination strategy could elicit a high and reliable protection against *Schistosoma japonicum* infection in mice. This report provides a basis for the development of transmission blocking veterinary vaccines in China.
